# Sucrose and Mannans Affect Arabidopsis Shoot Gravitropism at the Cell Wall Level

**DOI:** 10.3390/plants13020209

**Published:** 2024-01-11

**Authors:** Gregory Pozhvanov, Dmitry Suslov

**Affiliations:** 1 Department of Plant Physiology and Biochemistry, St. Petersburg State University, 199034 St. Petersburg, Russia; pozhvanov@binran.ru; 2 Laboratory of Analytical Phytochemistry, Komarov Botanical Institute of the Russian Academy of Sciences, 197376 St. Petersburg, Russia; 3 Department of Botany and Ecology, Herzen State Pedagogical University, 191186 St. Petersburg, Russia

**Keywords:** gravitropism, cell walls, sucrose, mannans, biomechanics, creep, metabolomics

## Abstract

Gravitropism is the plant organ bending in response to gravity. Gravitropism, phototropism and sufficient mechanical strength define the optimal position of young shoots for photosynthesis. Etiolated wild-type Arabidopsis seedlings grown horizontally in the presence of sucrose had a lot more upright hypocotyls than seedlings grown without sucrose. We studied the mechanism of this effect at the level of cell wall biomechanics and biochemistry. Sucrose strengthened the bases of hypocotyls and decreased the content of mannans in their cell walls. As sucrose is known to increase the gravitropic bending of hypocotyls, and mannans have recently been shown to interfere with this process, we examined if the effect of sucrose on shoot gravitropism could be partially mediated by mannans. We compared cell wall biomechanics and metabolomics of hypocotyls at the early steps of gravitropic bending in Col-0 plants grown with sucrose and mannan-deficient mutant seedlings. Sucrose and mannans affected gravitropic bending via different mechanisms. Sucrose exerted its effect through cell wall-loosening proteins, while mannans changed the walls’ viscoelasticity. Our data highlight the complexity of shoot gravitropism control at the cell wall level.

## 1. Introduction

The aboveground position of young plant shoots is tightly regulated to ensure efficient photosynthesis. It is determined by their gravi- and phototropism, as well as requiring sufficient mechanical strength to maintain the shoots’ own weight in the gravity field. Gravitropism is defined as the plant organ bending in response to gravity [1]. Plant shoots demonstrate negative gravitropism by bending upward after their inclination from the vertical [2]. Gravitropism consists of three sequential steps: gravity perception, gravity signal transduction and gravity response [3]. In shoots, gravity perception takes place in the endodermis [4]. The most popular explanation of gravity perception is the starch–statolith hypothesis, which involves the sedimentation of amyloplasts (starch-filled plastids) that function as statoliths to a new lower side in gravisensing cells (statocytes). After a series of signal transduction events, this results in the gravity response in the form of plant organ bending [3]. According to the Cholodny–Went theory, growth curvature is due to an unequal distribution of auxin between the two sides of the bending organ. Recent studies have uncovered signal transduction steps that link amyloplast sedimentation with auxin redistribution in plant gravitropism. LZY proteins translocate from sedimenting amyloplasts to the nearby plasmalemma in statocytes [5]. After that, they recruit RLD proteins from the cytoplasm to the plasmalemma [6]. Finally, the LZY-RLD module induces the polar relocalization of PIN3 [6], or different factors of auxin transport machinery [5], to form the IAA gradient in the gravistimulated organ.

Plant shoot gravitropism is an interesting experimental model because it involves simultaneous and well-coordinated changes in different physical cell wall properties, including its extensibility on the opposite sides of a gravistimulated organ [7], compression resistance on the concave side [1] and possibly flexibility of different cell wall components [8]. When studied in specific cell wall mutants and/or under specific treatments, plant shoot gravitropism may help reveal novel functions of cell wall polymers that are difficult to discover while studying the linear growth of plant organs. For example, novel functions have been established for xyloglucans [9] and mannans [8] using this model.

Sucrose plays a pivotal role in sugar transport, metabolism and signaling in plants [10]. It stimulates gravitropism at the stage of gravity perception by elevating the starch load in amyloplasts, which increases their weight and hence the rate of sedimentation [11,12]. Additionally, sucrose increases the proportion of upright hypocotyls in Arabidopsis seedlings grown on horizontal Petri plates [12]. In the present work, we examined if this effect of sucrose on hypocotyl posture was supported by mechanical and biochemical changes in cell walls. Sucrose was found to decrease the content of mannans in hypocotyl cell walls. We have recently demonstrated that these minor hemicelluloses interfere with the gravitropic bending of Arabidopsis hypocotyls [8]. Thus, we speculated that the stimulation of shoot gravitropism in the presence of sucrose could be partially mediated by mannans. Our detailed biomechanical and metabolomic analyses did not support this hypothesis. We found that sucrose and mannans controlled gravitropic bending via different mechanisms. This highlighted the complexity of shoot gravitropism regulation at the cell wall level.

## 2. Results

**Figure 1:**
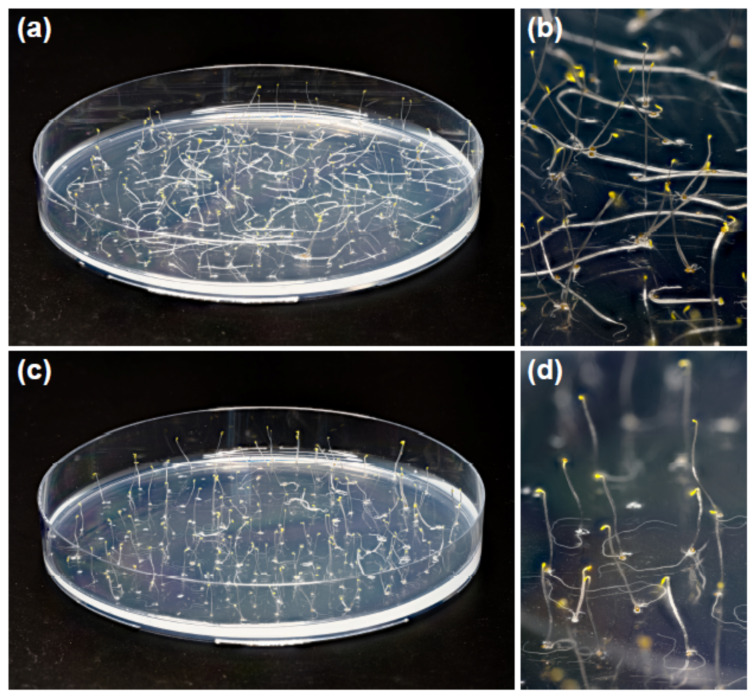
Sucrose in the growth medium increased the proportion of upright hypocotyls in etiolated Col-0 Arabidopsis seedlings. Five-day-old plants grown without sucrose (**a**,**b**) or in the presence of 1% *w*/*v* sucrose (**c**,**d**) on horizontal Petri plates.

Five-day-old etiolated Col-0 Arabidopsis seedlings grown on horizontal Petri plates on one-half-strength MS medium demonstrated different posture of hypocotyls depending on the presence of sucrose in the medium. We observed that about 56 ± 10% (mean ± SD) of control seedlings grown without sucrose had upright hypocotyls, while the hypocotyls of the remaining 44% lay on the agar surface (Figure 1a,b). Sucrose (1% *w*/*v*) supplementing the medium increased the proportion of upright hypocotyls to 98 ± 2% (Figure 1c,d). This difference in hypocotyl posture was highly significant (*p* = 0.0035; *n* = 4; Student’s *t*-test) and persisted from the moment of seed germination. Additionally, sucrose inhibited the growth of hypocotyls. It decreased their length to 11.8 ± 0.9 mm compared with 15.4 ± 2.5 mm in the control (mean ± SD; *n* = 40; *p* < 0.0001; Student’s *t*-test).

**Figure 2:**
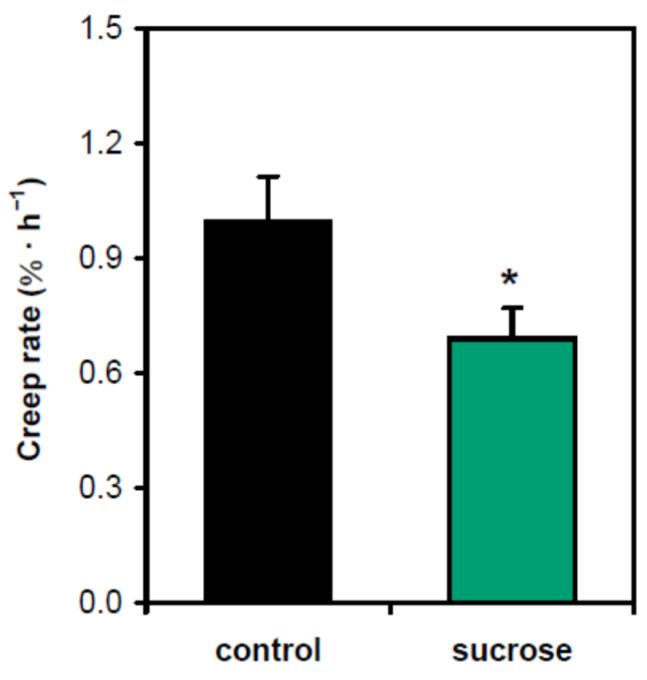
Sucrose strengthened the lower part of hypocotyls in five-day-old Arabidopsis seedlings grown on horizontal Petri plates. Five-millimeter-long basal segments of frozen/thawed heat-inactivated hypocotyls were extended at pH 5.0 under a 600 mg load, and their creep rate was measured. ‘Control’—plants grown without sucrose; ‘sucrose’—plants grown in the presence of sucrose (1% *w*/*v*). Data are means ± SE (*n* = 10). An asterisk denotes significant difference (*p* < 0.05; Student’s *t*-test).

The effect of sucrose on gravitropism has traditionally been linked to its involvement at the stage of gravity perception, where it increases the load of starch in amyloplasts [11,12]. However, our previous findings suggested that sucrose could also affect the posture of shoots via cell wall mechanics [12]. To test this option directly, we studied the creep of cell walls (i.e., their time-dependent deformation under a constant load) at the base of hypocotyls, the zone that carries the main part of the organ weight. We used heat inactivation of cell walls to eliminate any contribution of cell wall-loosening proteins to the biomechanics [13]. Sucrose significantly decreased the wall creep rate, which is consistent with the strengthening of the lower portion of hypocotyls (Figure 2).

**Figure 3:**
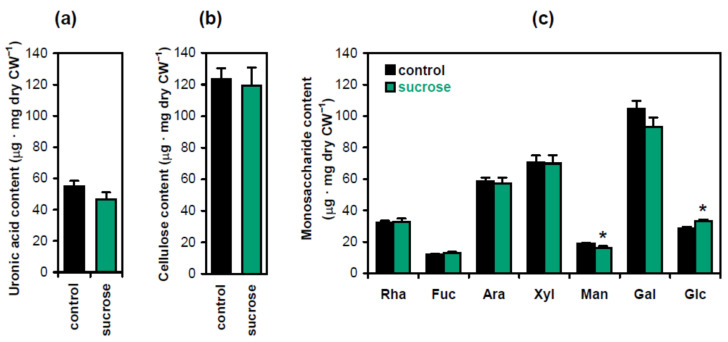
Biochemical composition of cell walls in Arabidopsis seedlings as affected by sucrose. The contents of uronic acids (**a**), crystalline cellulose (**b**) and monosaccharides derived from cell wall matrix polymers (**c**) were determined in five-day-old etiolated Col-0 seedlings grown on horizontal Petri plates without sucrose (control) or in the presence of sucrose (1% *w*/*v*). Data are means ± SE (*n* = 9). Asterisks denote significant differences (*p* < 0.05; Student’s *t*-test).

To investigate the biochemical basis of hypocotyl strengthening in the presence of sucrose, we carried out standard cell wall analyses measuring the levels of crystalline cellulose, uronic acids and monosaccharides derived from cell wall matrix polymers (Figure 3). Significant differences were only observed for monosaccharides: the content of glucose increased, which was accompanied by a decrease in mannose (Figure 3c). The increase in glucose was likely caused by starch. It accumulated in hypocotyls in the presence of sucrose [12] and could contaminate the wall residue, which was hydrolyzed with TFA (trifluoracetic acid) to release monosaccharides for subsequent analyses. The decrease in mannose suggested that the level of mannans was reduced in the presence of sucrose. These hemicelluloses have been known as the main source of mannose in Arabidopsis cell walls [14]. Their depletion is interesting in the context of shoot gravitropism. We have recently demonstrated that treatment with brassinazole, an inhibitor of brassinosteroid biosynthesis, and the triple mutation *csla2csla3csla9*, which both decreased mannans in Arabidopsis hypocotyl cell walls, stimulated gravitropic bending [8]. It might thus be hypothesized that sucrose, which also increased the gravitropic bending of Arabidopsis hypocotyls [12], could exert this effect, at least partially, at the expense of mannan depletion. We examined this hypothesis by studying the wall biomechanics and metabolomics of three-day-old Arabidopsis hypocotyls at the beginning of their gravitropic bending. Effects of sucrose in Col-0 plants were compared with those of the triple mutation *csla2csla3csla9*, which depleted mannans in Arabidopsis cell walls [14]. If the mechanism of sucrose action on gravitropic bending was partially mediated by mannans, we would observe similarities between the above two variants at the level of cell wall biomechanics and/or metabolomics.

**Figure 4:**
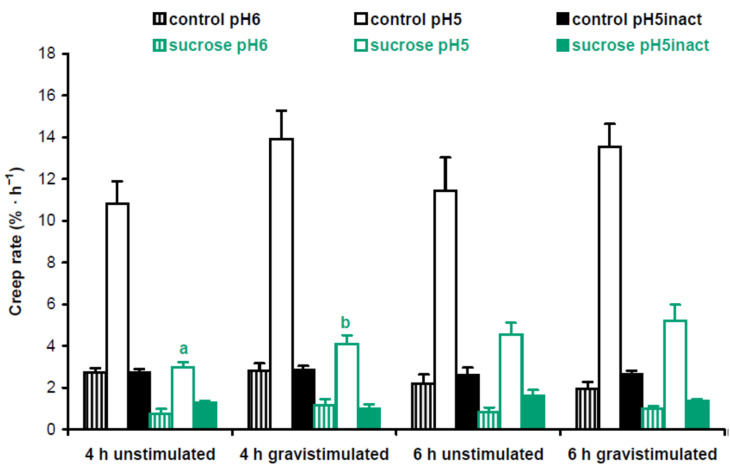
Cell wall biomechanics of hypocotyls from gravistimulated and nongravistimulated Col-0 Arabidopsis seedlings grown without sucrose (control) or in the presence of sucrose (1% *w*/*v*). Etiolated seedlings grown on vertical Petri plates for 3 days were either gravistimulated via a 90° counterclockwise rotation of the plates or left unstimulated. Both groups of seedlings were frozen 4 h and 6 h after the moment of gravistimulation. Three-millimeter-long subapical segments of frozen/thawed hypocotyls were extended under a 600 mg load, and their creep rate was measured. Data are means ± SE (*n* = 8). Different letters denote the only significant difference between gravistimulated and nongravistimulated hypocotyls (*p* < 0.05; Student’s *t*-test).

Cell wall mechanics were studied with the creep method using 3-mm-long subapical segments of hypocotyls from 3-day-old Arabidopsis seedlings grown on vertical Petri plates. We extended the zone of hypocotyls where their bending first appeared after gravistimulation. Cell wall creep was measured under three different conditions: (1) at pH 5, where expansin proteins, the mediators of ‘acid growth’, were active [15]; (2) at pH 6, where expansins were inactive, but different classes of cell wall-loosening proteins like XTHs (xyloglucan endotransglucosylase/hydrolases) could affect wall extensibility [16]; and (3) at pH 5 with heat inactivation, where activities of cell wall-loosening proteins were essentially eliminated, and in vitro extension of the wall was defined by its viscoelasticity [17]. Here ‘viscoelasticity’ denotes the range of material properties of cell walls that include elastic, viscous, plastic and retarded elastic deformations, but does not include enzyme-dependent, or chemorheological, flows [18]. To better understand the biomechanical mechanisms of gravitropic bending, we focused on comparisons between gravistimulated and nongravistimulated seedlings under each of the three conditions for creep measurements. In experiments with Col-0 plants grown with or without sucrose, we found only one significant difference in the creep rate between gravistimulated and nongravistimulated seedlings: at pH 5, 4 h after gravistimulation (Figure 4). This shows that increased gravitropic bending in the presence of sucrose [12] (Figure S1) could involve the accumulation of expansin proteins in the walls of hypocotyls.

**Figure 5:**
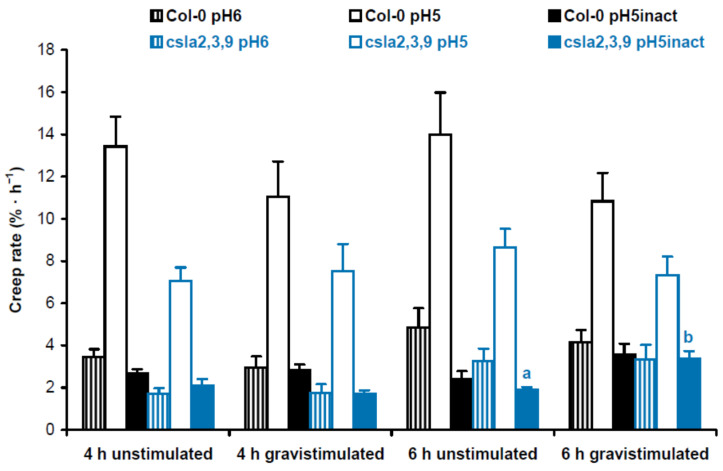
Cell wall biomechanics of hypocotyls from gravistimulated and nongravistimulated wild-type Col-0 Arabidopsis seedlings or mannan-deficient *csla2csla3csla9* triple mutants. Etiolated seedlings grown on vertical Petri plates for 3 days were either gravistimulated via a 90° counterclockwise rotation of the plates or left unstimulated. Both groups of seedlings were frozen 4 h and 6 h after the moment of gravistimulation. Three-millimeter-long subapical segments of frozen/thawed hypocotyls were extended under a 600 mg load, and their creep rate was measured. Data are means ± SE (*n* = 8). Different letters denote the only significant difference between gravistimulated and nongravistimulated hypocotyls (*p* < 0.05; Student’s *t*-test).

Five-day-old mannan-deficient *csla2csla3csla9* triple mutant and Col-0 wild-type seedlings grown on horizontal Petri plates did not differ in the percentage of upright hypocotyls (48.4 ± 5.8% vs. 47.7 ± 8.6%, respectively; mean ± SD; *n* = 7; *p* = 0.87; Student’s *t*-test). At the same time, the triple mutant demonstrated increased gravitropic bending of hypocotyls in three-day-old seedlings grown on vertical Petri plates (Figure S2) [8]. In biomechanical experiments where Col-0 wild-type plants were compared with *csla2csla3csla9* triple mutants, we also found only one significant difference in the creep rate between gravistimulated and nongravistimulated seedlings: at pH 5 with heat inactivation 6 h after gravistimulation (Figure 5). Thus, the increased gravitropic bending in the mannan-deficient triple mutant seemingly resulted from changes in wall viscoelasticity.

**Figure 6:**
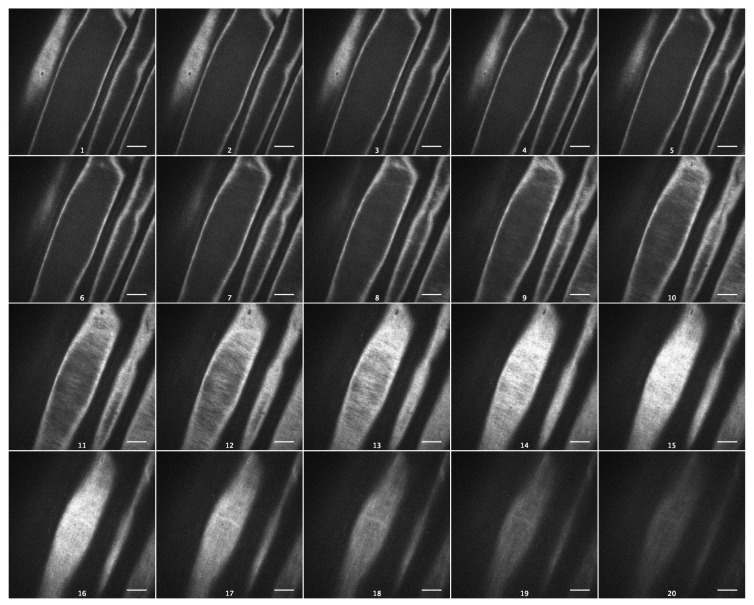
Cellulose macrofibril arrangement in the outer epidermal cell wall from the subapical part of five-day-old Col-0 hypocotyls. Spinning-disc confocal microscopy on wall samples stained with Pontamine Fast Scarlet 4B. Subsequent optical sections of a representative Z-stack from the innermost to the outermost wall layer. Scale bars are 10 μm.

**Figure 7:**
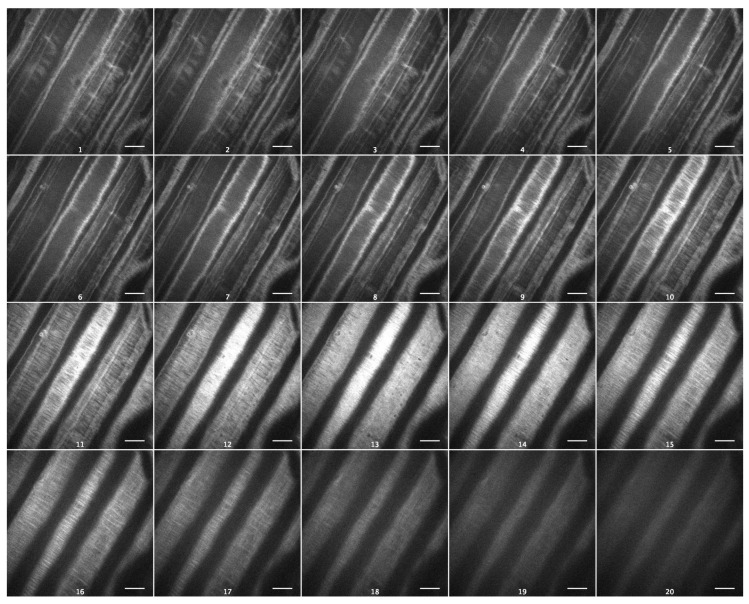
Cellulose macrofibril arrangement in the outer epidermal cell wall from the subapical part of five-day-old *csla2csla3csla9* hypocotyls. Spinning-disc confocal microscopy on wall samples stained with Pontamine Fast Scarlet 4B. Subsequent optical sections of a representative Z-stack from the innermost to the outermost wall layer. Scale bars are 10 μm.

As cellulose is the strongest cell wall component, with a profound effect on its viscoelasticity [19], we visualized cellulose macrofibrils in outer epidermal cell walls of Arabidopsis hypocotyls using spinning-disc confocal microscopy and the specific fluorescent dye Pontamine Fast Scarlet 4B (S4B) [20]. Cellulose macrofibrils were thin and predominantly transverse in the inner wall layers and longitudinal in the outer layers of Col-0 epidermal cell walls (Figure 6, Video S1). In contrast to this, they were thicker and more uniformly transversely orientated in the inner wall layers of *csla2csla3csla9* hypocotyls (Figure 7, Video S2). Thus, the depletion of mannans altered the arrangement of cellulose macrofibrils in the triple mutant, which could explain the effect on wall creep (Figure 5).

Overall, our biomechanical data demonstrated that the stimulation of shoot gravitropism with sucrose (Figure 4) and in the triple mutant (Figure 5) occurred via different mechanisms. This does not support our hypothesis that the effect of sucrose on gravitropism is partially mediated by mannans in Arabidopsis hypocotyls.

In our metabolomic studies, we used Arabidopsis seedlings grown and gravistimulated under the same conditions and schedule as reported for biomechanical experiments (Figure 4 and Figure 5), but the plant material was harvested for analysis only at one timepoint: 4 h after gravistimulation. Only those metabolites that were present in all eight biological replicates (three independent experiments) were considered.

**Figure 8:**
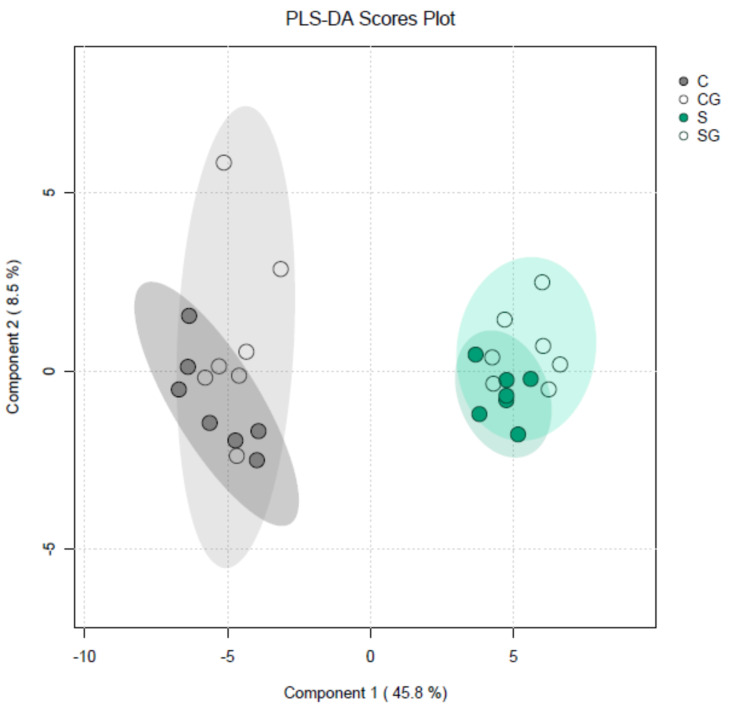
Partial least squares discriminant analysis (PLS-DA) of metabolite profiles of Arabidopsis hypocotyls. Three-day-old etiolated Col-0 seedlings grown on vertical Petri plates without sucrose or in the presence of sucrose (1% *w*/*v*) were gravistimulated via a 90° counterclockwise rotation of the plates. PLS-DA scores are plotted along the X and Y axes, which are Component 1 and Component 2, respectively. Percentage in brackets indicates the explained variance of metabolite content. Abbreviations: C—nongravistimulated wild-type Col-0 seedlings, grown without sucrose; S—nongravistimulated wild-type Col-0 seedlings, grown in the presence of sucrose (1% *w*/*v*); CG—gravistimulated wild-type Col-0 seedlings, grown without sucrose; SG—gravistimulated wild-type Col-0 seedlings, grown in the presence of sucrose (1% *w*/*v*). Each circle shows a corresponding biological replicate. Large ellipses depict 95% confidence regions.

Sucrose induced profound metabolic changes in Arabidopsis hypocotyls. We found significant differences for 47 metabolites out of 60 detected in each experimental variant that were caused by the presence of sucrose in the growth medium (Table S1, comparisons of C vs. S and CG vs. SG). The effect of sucrose was very specific for particular metabolites: it increased and decreased the contents of 20 and 27 metabolites, respectively. The group of metabolites whose content decreased in the presence of sucrose included various amino acids. Apparently, this indicates that sucrose had a strong stimulatory effect on protein synthesis. At the same time, the group of metabolites whose content was higher in the presence of sucrose included a number of sugars. Interestingly, the effects of sucrose in the nongravistimulated variants (C vs. S) usually matched those in the gravistimulated variants (CG vs. SG). At the same time, we found only two metabolites: hydroxybutyric acid and ornithine, the content of which changed significantly as a result of gravistimulation in the presence of sucrose (S vs. SG). This means that sucrose’s effect on metabolism could outweigh and mask the majority of fine metabolic changes caused by gravistimulation. The plot of partial least squares discriminant analysis (PLS-DA) of metabolite profiles of Arabidopsis hypocotyls (Figure 8) illustrated this conclusion. The ellipses describing gravistimulated (SG) and nongravistimulated (S) plants grown with sucrose were not separated.

**Figure 9:**
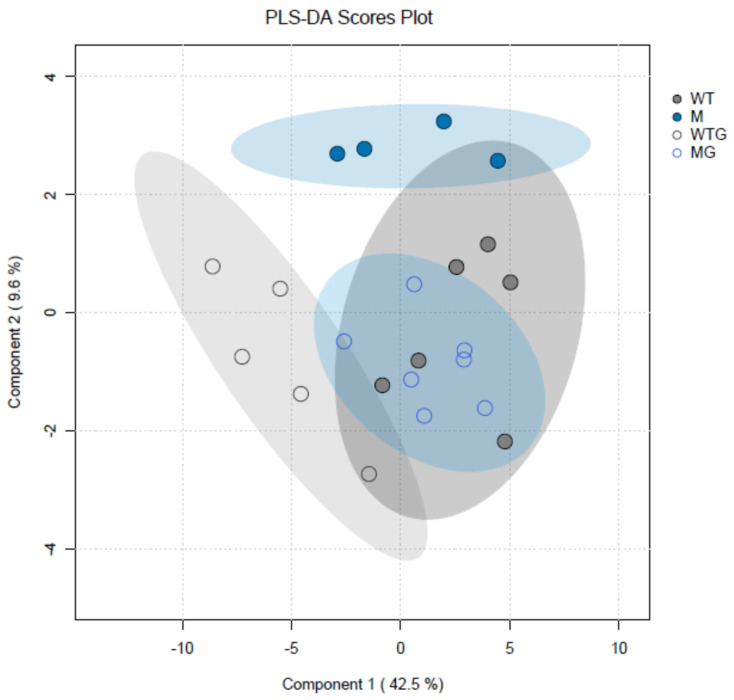
Partial least squares discriminant analysis (PLS-DA) of metabolite profiles of wild-type Col-0 and *csla2csla3csla9* Arabidopsis hypocotyls. Three-day-old etiolated wild-type Col-0 and *csla2csla3csla9* seedlings grown on vertical Petri plates without sucrose were gravistimulated via a 90° counterclockwise rotation of the plates. PLS-DA scores are plotted along X and Y axes that are Component 1 and Component 2, respectively. Percentage in brackets indicate the explained variance for metabolite content. Abbreviations: WT—nongravistimulated wild-type Col-0 seedlings; M—nongravistimulated *csla2csla3csla9* triple mutant seedlings; WTG—gravistimulated wild-type Col-0 seedlings; MG—gravistimulated *csla2csla3csla9* triple mutant seedlings. Each circle shows a corresponding biological replicate. Large ellipses depict 95% confidence regions.

Metabolomic analyses of *csla2csla3csla9* triple mutants vs. wild-type Col-0 plants gave very different results (Table S2) compared with the effects of sucrose (Table S1). Pairwise comparisons of the four experimental variants revealed only eight significant differences for the content of metabolites. Remarkably, seven of them were found when gravistimulated mutant and wild-type plants were compared (WTG vs. MG). Thus, gravistimulation caused stronger metabolic changes in the triple mutants compared with Col-0 plants (Figure 9). A decrease in 4-hydroxyproline and an increase in aspartic acid in gravistimulated *csla2csla3csla9* seedlings could suggest that gravistimulation activated the metabolism of cell wall structural glycoproteins.

By analogy with the data on biomechanics (Figure 4 and Figure 5), the results of metabolomic analyses did not reveal any appreciable similarity in the effects of mannan deficiency and sucrose. Therefore, our hypothesis that the effect of sucrose on shoot gravitropism could be partially mediated by mannans was not supported.

## 3. Discussion

In the present work, we found that the effect of sucrose on hypocotyl posture (Figure 1c,d) was only partially due to improved gravity perception [11,12]. Sucrose also acted at the cell wall level: (1) it strengthened hypocotyls at the base (Figure 2), which aided them in supporting their own weight in the gravity field; (2) it accelerated gravitropic bending by an expansin-mediated mechanism (Figure 4).

Interestingly, the increase in hypocotyl strength was accompanied by minor changes in wall composition (Figure 3). Apparently, strengthening was mainly achieved through an increase in the amount of cell wall material, rather than through the accumulation of stronger wall material. Indeed, the rate of cell wall synthesis in Arabidopsis hypocotyls, estimated through the rate of cellulose synthase complex trafficking in the plasmalemma, was controlled by the availability of sugars from photosynthesis or the growth medium [13]. The increase in cellulose synthesis in the presence of sucrose [13] was seemingly accompanied by a comparable stimulation in the synthesis of all main cell wall constituents, with the exception of mannans, which was reflected in the limited sucrose-induced changes in the wall composition (Figure 3). In line with this assumption, we found that sucrose decreased the contents of arginine, asparagine, valine, glycine, isoleucine, leucine, proline, serine, threonine, tryptophan, phenylalanine and uronic acids (Table S1). A significant part of these amino acids might have been used in the synthesis of cell wall structural glycoproteins, while phenylalanine and uronic acids could have been consumed in the synthesis of cell wall phenylpropanoid compounds and pectins, respectively.

The in vivo strength of young plant organs is defined by turgor [21]. Both stronger cell walls and higher intracellular concentrations of osmotically active compounds increase turgor [22]. The higher proportion of upright hypocotyls in the presence of sucrose (Figure 1) could also result from elevated turgor due to the accumulation of intracellular osmotics. Our metabolomics data supported this additional mechanism of sucrose action on the hypocotyl posture: many abundant osmotically active compounds accumulated in hypocotyls in the presence of sucrose including arabinose, glucose, mannose, sucrose and others (Table S1).

Lastly, the inhibition of hypocotyl growth observed in the presence of sucrose, resulting from delayed germination of Arabidopsis seeds [23], could also contribute to the higher proportion of upright hypocotyls (Figure 1) because shortened hypocotyls would be more stable on a horizontal surface.

The only change in cell wall biochemical composition observed in the presence of sucrose was a decrease in mannans (Figure 3). Mannan deficiencies have been shown to disturb the arrangement of cellulose in Arabidopsis [24,25]. As cellulose is critical for wall strength [19], this change in mannans would hardly increase the percentage of upright hypocotyls. We found here that mannan depletion did not contribute to the control of hypocotyl posture, as the *csla2csla3csla9* triple mutant and Col-0 wild-type seedlings had the same percentage of upright hypocotyls.

Gravitropic bending results from differential cell growth on the opposite sides of gravistimulated organs [26]. In elongating shoots placed horizontally, growth is always inhibited on the upper side [27]. In some plant species, this inhibition is accompanied by growth acceleration on the lower side of gravistimulated shoots [7,28,29]. The differential growth response is controlled by the corresponding decrease and increase in the wall extensibility, and these opposite changes were not necessarily equal in magnitude and synchronous in time on both sides of gravistimulated shoots [7,28]. The Arabidopsis hypocotyls used in the present study were very small and fragile. The longitudinal bisection of gravistimulated Arabidopsis hypocotyls into equal upper and lower halves to study their biomechanics separately is very difficult. Thus, we used nonbisected Arabidopsis hypocotyls to measure their wall properties during gravitropism. The reported wall creep rates (Figure 4 and Figure 5) were the net estimation of probable opposite changes in wall extensibility on upper and lower sides of gravistimulated hypocotyls. One limitation of our approach is that any significant differences between gravistimulated and nongravistimulated hypocotyls could be revealed only if the changes on both sides of the former were different in magnitude and/or timing.

Our previous data suggested that a decrease in mannans could stimulate gravitropic bending [8]. Therefore, it may be a part of the mechanism of the increased shoot gravitropism in the presence of sucrose [12] (Figure S1). However, this assumption was not supported by our biomechanical (Figure 4 and Figure 5) and metabolomic analyses (Figure 8 and Figure 9, Tables S1 and S2). We found that the increased gravitropic bending of hypocotyls in sucrose-grown plants (Figure S1) was accompanied by increased in vitro cell wall extension at pH 5 (Figure 4). This is consistent with the accumulation of expansin proteins, the mediators of the acid growth known as the early phase of auxin-induced plant organ extension [15,30,31]. Interestingly, the plasmalemma H^+^-ATPases were activated in the lower part of gravistimulated hypocotyls of Arabidopsis plants grown with sucrose [32]. They acidified the apoplast in this part of the hypocotyls, shifting its pH to optimal values for expansin activities [32]. These data suggest that the increased sucrose-induced gravitropic bending (Figure S1) was based on the enhanced auxin response, in which H^+^-ATPases and expansins worked in a concerted fashion. Apparently, heavier amyloplasts accumulated in the presence of sucrose [12] established a steeper auxin gradient in Arabidopsis hypocotyls after their gravistimulation.

The effect of mannans on gravitropic bending (Figure S2) [8] (p. 687) had a different mechanism compared with the effect of sucrose. It was accompanied by a significant increase in the creep rate of heat-inactivated cell walls (Figure 5). This shows that the wall viscoelasticity was changed as a result of mannan deficiency in the *csla2csla3csla9* triple mutant. One mechanism for this change may involve cellulose macrofibrils that became thicker and more transversally orientated in mannan-deficient cell walls (Figure 6 and Figure 7; Videos S1 and S2) in the zone of hypocotyls where their gravitropic bending normally develops. Mannans were reported to interact with the surface of cellulose microfibrils [24,33] and may affect cellulose organization [33]. Our findings (Figure 6 and Figure 7) could be explained by the more abundant lateral interactions between the adjacent cellulose microfibrils in the absence of mannans, resulting in the formation of thicker bundles (Figure 7, Video S2). Additionally, mannan deficiency may expose more sites for the specific S4B binding on the surface of cellulose macrofibrils, such that they look brighter. A number of indirect data suggest that cellulose microfibrils considerably reoriented in the course of gravitropic bending [34,35,36,37]. One may hypothesize that mannan depletion eliminates steric barriers for these microfibril reorientations, which facilitates the gravity response.

The second mechanism for the effect of mannans on gravitropic bending could involve cell wall structural glycoproteins. We demonstrated that gravistimulation of the triple mutant resulted in a decrease in the content of 4-hydroxyproline and a simultaneous increase in the content of aspartic acid (Table S2). Hydroxyproline is a hallmark amino acid residue of cell wall structural glycoproteins, including extensins, that form a rigid network in the cell wall, decreasing its extensibility through the formation of isodityrosine cross-links [38,39]. Aspartic acid is an essential biosynthetic precursor for tyrosine, another abundant amino acid residue in extensins [40]. Thus, gravistimulation of the triple mutant could modulate the metabolism of extensin proteins in Arabidopsis hypocotyl cell walls. A possible link between mannans and extensins is illustrated in Figure S3. It is based on the fact that ascorbate and mannans have GDP-D-mannose as a common biosynthetic precursor [41], while ascorbate is an essential cofactor in the posttranslational synthesis of hydroxyproline from proline which is catalyzed by prolyl 4-hydroxylase enzymes [41,42]. Thus, ascorbate and mannan biosynthesis pathways could compete for the common biosynthetic precursor.

The third potential mechanism by which mannans could affect gravitropic bending is also mediated by ascorbate, a potent scavenger of reactive oxygen species (ROS) [41]. ROS are important in the early stages of shoot and root gravitropism, where they presumably have a role in signaling [43,44,45,46]. Thus, their scavenging by ascorbate could affect gravitropism, while the level of ascorbate, in turn, could be modulated by changes in mannan biosynthesis (Figure S3).

Further studies are needed to understand the mechanism underpinning the effect of mannans on shoot gravitropism.

To sum up, mannans did not mediate the stimulatory effect of sucrose on shoot gravitropism. Mannans and sucrose influenced the gravitropic bending of Arabidopsis hypocotyls through distinct cell wall-related mechanisms. Sucrose exerted its effect via the cell wall-loosening proteins expansins, while the effect of mannans was based on changes in wall viscoelasticity. This highlights the complexity of shoot gravitropism control at the cell wall level.

## 4. Materials and Methods

### 4.1. Plant Materials and Growth Conditions

*Arabidopsis thaliana* (L. Heynh.) wild-type Columbia-0 and *csla2csla3csla9* mutant plants were grown on half-strength Murashige and Skoog (MS) medium (pH 5.7) (Duchefa Biochemie, Haarlem, the Netherlands) containing 0.68% (*w*/*v*) microagar (Duchefa Biochemie) and, where indicated, 1% (*w*/*v*) sucrose (Sigma-Aldrich, St. Louis, MO, USA).

Surface-sterilized seeds were sown aseptically on the above-mentioned medium on 145 mm × 20 mm round Petri plates (Greiner Bio-One, Mosonmagyaróvár, Hungary) for experiments with plants grown on horizontal plates or 120 mm × 120 mm × 17 mm square Petri plates (Greiner Bio-One) for experiments with plants grown on vertical plates. The seeds were stratified for 2 days at 4 °C, and their synchronous germination was induced via exposure to white fluorescent light (150 μmol m^−2^ s^−1^) for 6 h at 21 °C. The moment of transfer to light was taken as zero age for experimental plants. After the light induction period, the Petri plates were wrapped in two layers of thick aluminum foil, placed horizontally or vertically in a growth chamber (ShSV-132 P, Termocon, St. Petersburg, Russia) at 21 °C and kept there for 3 or 5 days. Gravitropism in experimental vertical Petri plates with three-day-old seedlings was induced via a 90° counterclockwise rotation of the plates (gravistimulation), and the seedlings were harvested for analysis 4 h and 6 h after their gravistimulation. Control vertical Petri plates with three-day-old seedlings were not rotated, and these nongravistimulated plants were harvested at the same age (76 h and 78 h) as their gravistimulated counterparts.

### 4.2. Hypocotyl Length and Curvature Determination

Arabidopsis seedlings were photographed with a digital camera (Canon SX510 HS, Zhuhai, China). Hypocotyl length and gravitropic bending were determined on the photos obtained using the ‘Segmented line’ and ‘Angle’ tools in ImageJ 1.49b software (NIH, Bethesda, MD, USA), respectively.

### 4.3. Cell Wall Biochemical Analyses

Whole five-day-old etiolated Arabidopsis seedlings with their seeds removed were used in biochemical analysis of cell walls as detailed in [8].

### 4.4. Extensometry

Arabidopsis seedlings for creep tests were placed individually into 1.5-mL Eppendorf test tubes, frozen by immersing the closed tubes into liquid nitrogen, stored at −20 °C and used for biomechanical analyses within 2 weeks after freezing. In vitro extension of frozen/thawed hypocotyls was measured with a custom-built constant-load extensometer [47,48]. A 5-mm-long basal segment (located 1.5 mm above the root neck) of a five-day-old hypocotyl or a 3-mm-long subapical segment (located 1.5 mm below the apical hook) of a 76(78)-hour-old hypocotyl was secured between clamps of an extensometer and preincubated in a buffer (20 mM sodium acetate, pH 5.0, or 20 mM MES-KOH, pH 6.0) in a relaxed state for 2 min. Then, its time-dependent extension (creep) was measured in the same buffer under a 600 mg load for 15 min. The relative creep rate was calculated as described in [12]. Before some creep tests, hypocotyl cell walls were heat-inactivated at 90 °C for 3 min as described in [17].

### 4.5. Spinning-Disc Confocal Microscopy

Cellulose macrofibrils were visualized in cell walls with Pontamine Fast Scarlet 4B (S4B) dye [20]. To improve the dye penetration into the cell walls of Arabidopsis hypocotyls, plants were extracted under mild conditions via sequential washes with EtOH:100% acetic acid (7:1, *v*/*v*) for 1 h; 100% EtOH for 15 min; 50% EtOH in H_2_O for 15 min; and 1M KOH in H_2_O for 30 min. All washing steps were carried out on a rotator. Experimental samples were stored in 1M KOH at 4 °C before analysis or used immediately after the last alkaline wash for cellulose visualization. The samples were rinsed in H_2_O before staining to remove residual KOH, after which 0.2% (*w*/*v*) S4B was added for 30 min. Then they were rapidly rinsed with a large volume of H_2_O to remove excess dye and observed under a Nikon Ti-E inverted confocal microscope equipped with a CSU-X1 spinning disc head (Yokogawa, Musashino, Japan), a 100× CFI Apo oil immersion TIRF objective (NA 1.49, Nikon, Tokyo, Japan), an evolve charge-coupled device camera (Photometrics Technology, Tucson, AZ, USA), and a 31.2× lens between the spinning disc and camera. S4B was excited using a 561 nm laser (similar to [20]). Image acquisitions were performed using Metamorph software (Molecular Devices, San Jose, CA, USA) version 7.5. Corresponding Z-stacks were visualized in Fiji 2.0.0-rc-69 (ImageJ 1.52p) as a series of subsequent optical sections using the ‘Make Montage’ function or exported as movies at a frequency of 7 frames per second.

### 4.6. Metabolomics

Arabidopsis hypocotyls were harvested from Petri plates (from 50 to 120 plants per sample, plants from one Petri plate were combined into one biological replicate), dissected from their roots, put into screw-cap 2 mL test tubes (SSI, Lodi, CA, USA) and immediately frozen in liquid nitrogen, then stored at −80 °C. Frozen hypocotyls were then ground at the temperature of liquid nitrogen in pre-cooled holders in a Retsch MM 400 ball vibration mill (Haan, Germany) at 27 Hz for 3 min with one 2 mm steel ball per test tube. Polar metabolites were then extracted in methanol with ribitol as an internal standard and dried to a thin film in a Labconco Centrivap vacuum centrifuge (Kansas City, MO, USA) at +4 °C and stored at −80 °C. Metabolites were then chemically derivatized in two steps (methoxyamination and silylation) [49]. Chromatography analysis was performed on a Leco Pegasus 4D GC × GC TOFMS equipped with a Gerstel autosampler on a Zebron ZB-5MS column (5% phenylmethylsiloxane, 30 m length, 250 μm internal diameter, 0.25 μm film thickness; Phenomenex, Torrance, CA, USA). Metabolite identification and their quantification was performed in MS-DIAL v. 4.8 software [50] using the Golm Metabolome database [51] and Fiehn BinBase [52]. Final matrices containing information on relative metabolite content were analyzed in Metaboanalyst [53].

### 4.7. Statistical Analysis

All data reported were based on two to three independent experiments, and the total number of biological replicates was indicated in the respective figures and tables. One biological replicate in different assays was as follows: about 120 seedlings grown on one Petri plate for hypocotyl posture determination; individual Arabidopsis seedlings grown on at least four different Petri plates for hypocotyl length, gravitropic bending and creep rate measurements; about 300 seedlings from one Petri plate with their seeds removed for cell wall biochemical analyses; and hypocotyls from 50–120 seedlings grown on one Petri plate for metabolomics. Means and SD(SE) values were calculated on the basis of the above biological replicates. Statistical differences between the means were revealed using Student’s *t*-tests, and the difference was considered as significant at *p* < 0.05.

## Data Availability

The data presented in this study are available on request from the corresponding author. The data are not publicly available due to privacy restrictions.

## Supplementary Materials

The following supporting information can be downloaded at: https://www.mdpi.com/article/10.3390/plants13020209/s1, Figure S1: Sucrose increases the gravitropic bending of hypocotyls in Arabidopsis plants; Figure S2: Mannan-deficient *csla2csla3csla9* triple mutants of Arabidopsis plants demonstrate increased gravitropic bending of hypocotyls; Figure S3: Biosynthesis pathways explain why the cell wall levels of mannans and extensins could be interdependent; Table S1: Relative content of metabolites in hypocotyls of gravistimulated and nongravistimulated wild-type Col-0 Arabidopsis seedlings, grown without sucrose (control) or in the presence of sucrose (1% *w*/*v*) in the growth medium; Table S2: Relative content of metabolites in hypocotyls of gravistimulated and nongravistimulated wild-type Col-0 and *csla2csla3csla9* Arabidopsis seedlings; Video S1: Cellulose macrofibril arrangement from the innermost to the outermost layer of the outer epidermal cell wall from the subapical part of five-day-old Col-0 hypocotyls. Video S2: Cellulose macrofibril arrangement from the innermost to the outermost layer of the outer epidermal cell wall from the subapical part of five-day-old *csla2csla3csla9* hypocotyls.
