# Supplementary material for: Sucrose and Mannans Affect Arabidopsis Shoot Gravitropism at the Cell Wall Level
Source: Plants (Basel). 2024 Jan 11;13(2):209. doi: 10.3390/plants13020209 (PMC10819476; doi:10.3390/plants13020209)
Supplement: Supplementary file 1 [file plants-13-00209-s001.zip › Figure S1.pdf]

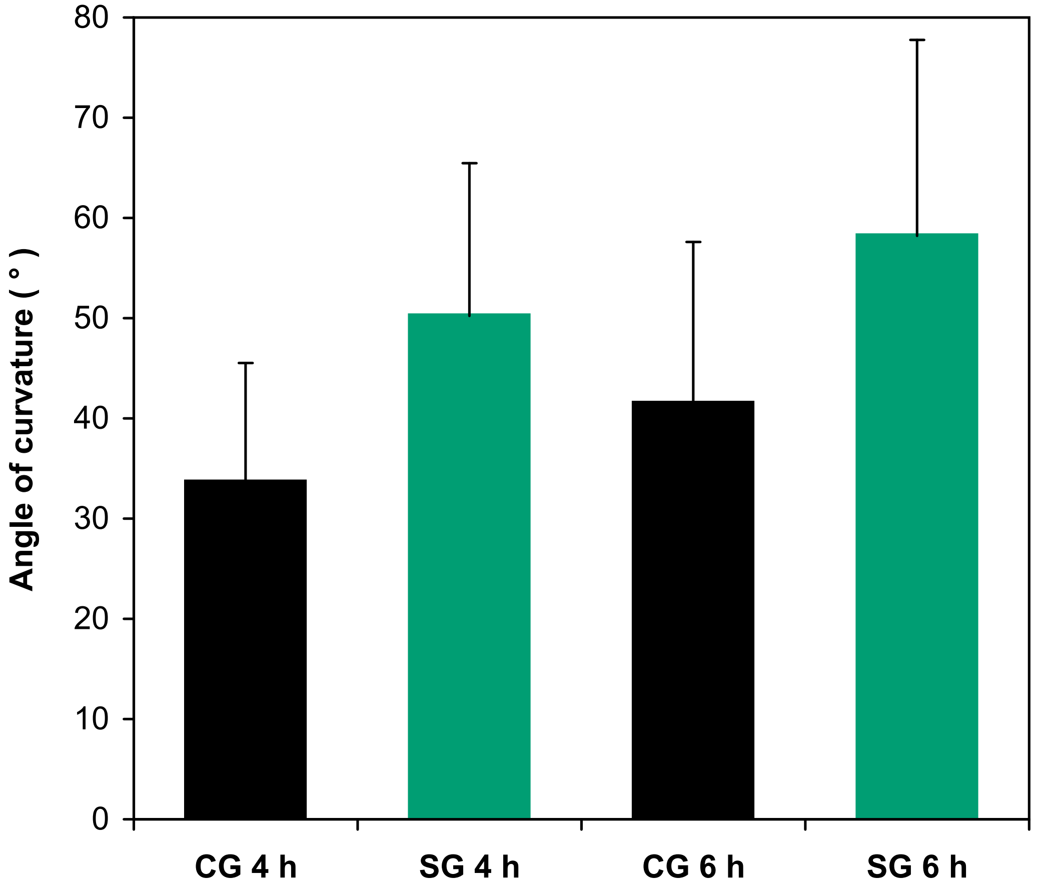

Figure S1. Sucrose increases the gravitropic bending of hypocotyls in Arabidopsis plants. Three-day-old etiolated Col-0 seedlings grown on vertical Petri plates without sucrose or in the presence of sucrose (1% w/v) were gravistimulated by a 90° counterclockwise rotation of the plates. Angles of their hypocotyl curvature were measured 4 h and 6 h after the moment of gravistimulation. Abbreviations: CG - gravistimulated plants grown without sucrose; SG - gravistimulated plants grown in the presence of sucrose. Data are means ± SD (n=80). Sucrose significantly (P<0.0001, Student's t-test) increased the angle of hypocotyl bending 4 h and 6 h after gravistimulation.
