# Supplementary material for: Sucrose and Mannans Affect Arabidopsis Shoot Gravitropism at the Cell Wall Level
Source: Plants (Basel). 2024 Jan 11;13(2):209. doi: 10.3390/plants13020209 (PMC10819476; doi:10.3390/plants13020209)
Supplement: Supplementary file 1 [file plants-13-00209-s001.zip › Figure S2.pdf]

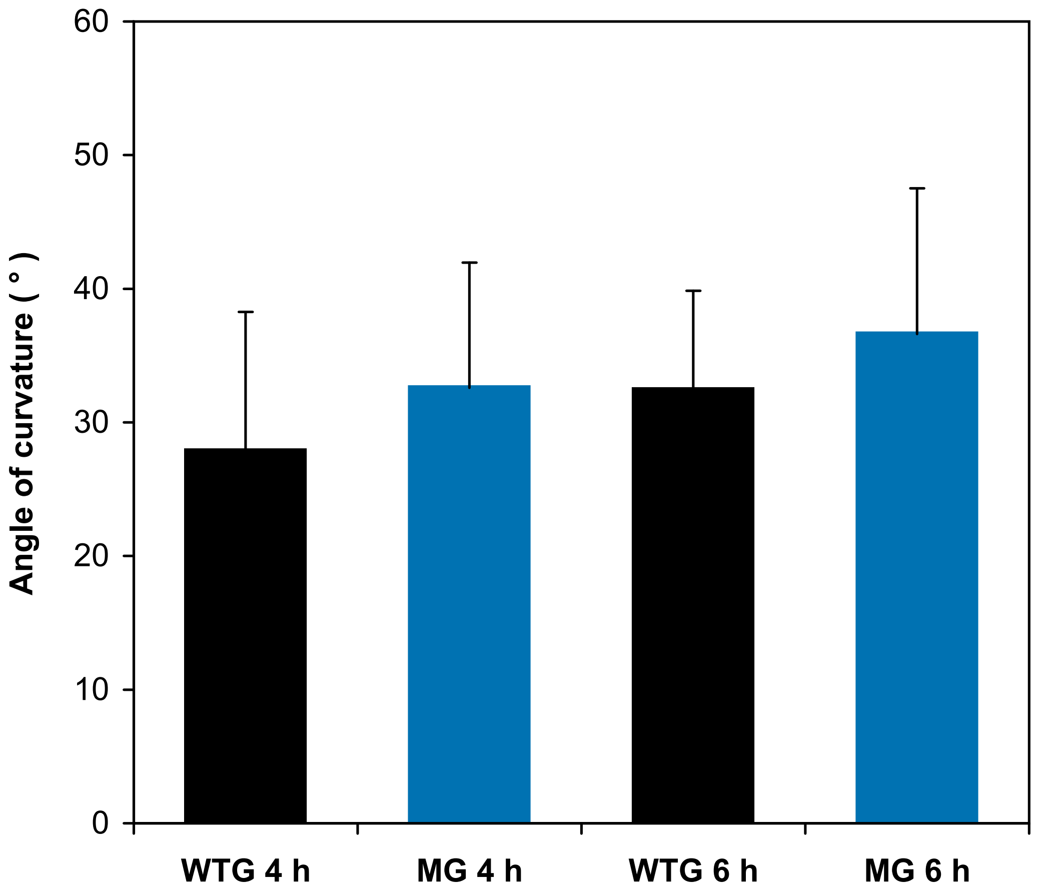

Figure S2. Mannan-deficient *csla2csla3csla9* triple mutants of Arabidopsis plants demonstrate increased gravitropic bending of hypocotyls. Three-day-old etiolated Col-0 and *csla2csla3csla9* seedlings grown on vertical Petri plates were gravistimulated by a 90° counterclockwise rotation of the plates. Angles of their hypocotyl curvature were measured 4 h and 6 h after the moment of gravistimulation. Abbreviations: WTG - gravistimulated Col-0 plants; MG - gravistimulated *csla2csla3csla9* triple mutant plants. Data are means ± SD (n=45). The triple mutation significantly ($P<0.05$, Student's t-test) increased the angle of hypocotyl bending 4 h and 6 h after gravistimulation.
