# Supplementary material for: Sucrose and Mannans Affect Arabidopsis Shoot Gravitropism at the Cell Wall Level
Source: Plants (Basel). 2024 Jan 11;13(2):209. doi: 10.3390/plants13020209 (PMC10819476; doi:10.3390/plants13020209)
Supplement: Supplementary file 1 [file plants-13-00209-s001.zip › Figure S3.pdf]

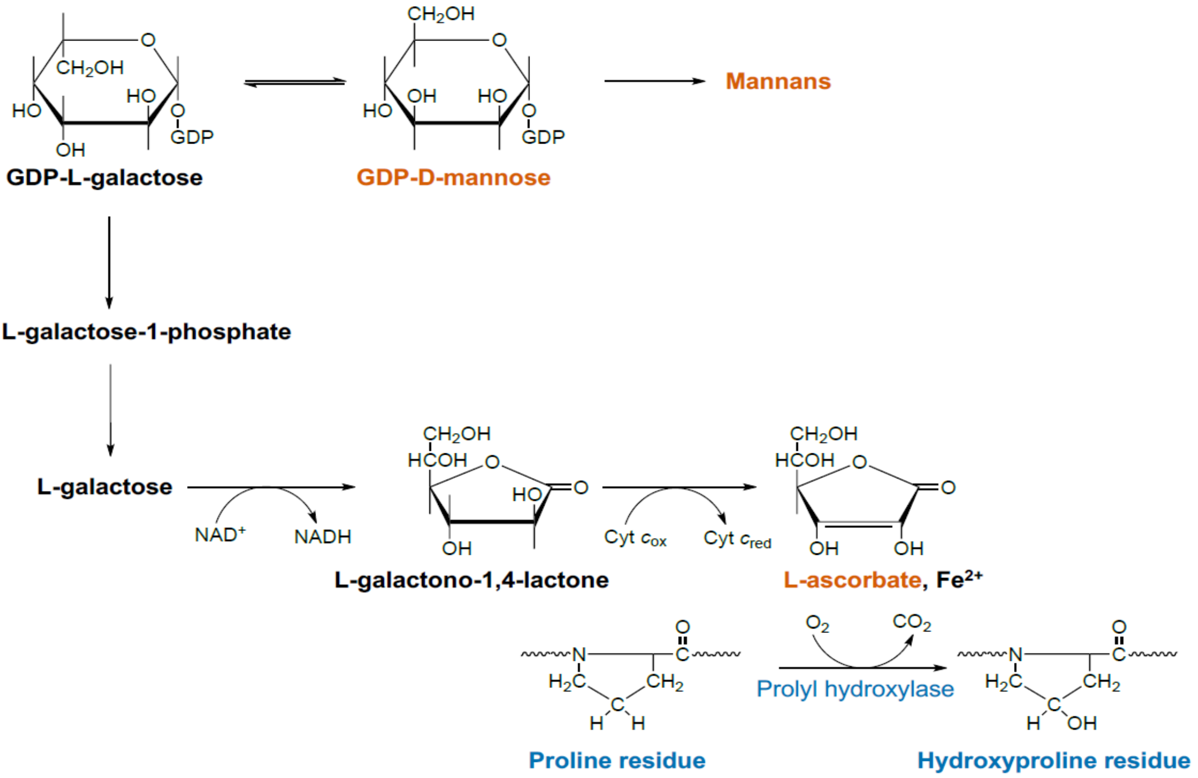

Figure S3. Biosynthesis pathways explain why the cell wall levels of mannans and extensins could be interdependent. GDP-D-mannose is a common biosynthetic precursor for mannans and ascorbate. Ascorbate is an essential cofactor of prolyl hydroxylase enzymes that posttranslationally hydroxylate proline residues of cell wall structural glycoproteins to yield hydroxyproline residues.
