# Supplementary material for: Sucrose and Mannans Affect Arabidopsis Shoot Gravitropism at the Cell Wall Level
Source: Plants (Basel). 2024 Jan 11;13(2):209. doi: 10.3390/plants13020209 (PMC10819476; doi:10.3390/plants13020209)
Supplement: Supplementary file 1 [file plants-13-00209-s001.zip › Table S1.pdf]

Table S1. Relative content of metabolites in hypocotyls of gravistimulated and nongravistimulated wild type Col-0 Arabidopsis seedlings, grown without sucrose (control) or in the presence of sucrose (1% w/v) in the growth medium. Results of three independent experiments (n=8). Data are given in arbitrary units.
Abbreviations:
C - nongravistimulated wild type Col-0 seedlings, grown without sucrose;
S - nongravistimulated wild type Col-0 seedlings, grown in the presence of sucrose (1% w/v);
CG - gravistimulated wild type Col-0 seedlings, grown without sucrose;
SG - gravistimulated wild type Col-0 seedlings, grown in the presence of sucrose (1% w/v).
Data are means, standard errors (SE) and *P* values of pairwise comparisons of the above variants using Student's *t*-test.
All metabolites for which significant differences between nongravistimulated and gravistimulated variants (C vs. CG and S vs. SG) were found are indicated in bold. In the case of a significant increase in the content of a metabolite in gravistimulated vs. nongravistimulated seedlings (CG vs. C and SG vs. S), as well as in seedlings grown in the presence of sucrose compared with those grown without sucrose (S vs. C and SG vs. CG) the respective *P* values are shown on a red background. In the case of a significant decrease in the content of a metabolite in the abovementioned comparisons the respective *P* values are shown on a blue background.

Metabolite	C	C, SE	CG	CG, SE	S	S, SE	SG	SG, SE	*P* value, C/CG	*P* value, C/S	*P* value, CG/SG	*P* value, S/SG
Alanine	1.066	0.062	1.330	0.212	2.189	0.290	2.446	0.487	0.132	0.004	0.032	0.330
**γ-Aminobutyric acid**	0.401	0.025	0.301	0.043	0.690	0.044	0.841	0.108	0.036	0.000	0.001	0.114
Arabinose	0.451	0.084	0.384	0.056	1.564	0.045	1.596	0.175	0.261	0.000	0.000	0.432
**Arginine**	0.620	0.035	0.490	0.042	0.209	0.028	0.236	0.041	0.017	0.000	0.000	0.295
Ascorbic acid	21.138	14.903	16.324	10.814	103.568	5.786	102.180	18.642	0.399	0.000	0.001	0.472
Asparagine	3.878	0.709	3.782	0.684	0.518	0.080	0.531	0.120	0.462	0.002	0.001	0.467
**Asparagine, dehydro-**	1.021	0.104	0.622	0.116	0.167	0.014	0.175	0.027	0.012	0.000	0.003	0.399
**Aspartic acid**	1.190	0.084	0.851	0.155	1.388	0.058	1.617	0.131	0.041	0.040	0.001	0.071
N-acetylglucosamine	13.949	1.658	11.829	2.008	8.306	0.588	9.417	1.616	0.215	0.007	0.183	0.267
N-acetylglucosamine 6-phosphate	0.034	0.007	0.028	0.007	0.189	0.023	0.224	0.035	0.292	0.000	0.000	0.209
Benzoic acid	0.074	0.007	0.074	0.015	0.074	0.007	0.072	0.014	0.497	0.488	0.477	0.459
β-Alanine	0.758	0.055	0.645	0.094	0.639	0.034	0.742	0.115	0.162	0.048	0.263	0.207
Valine	2.683	0.252	2.131	0.355	0.327	0.014	0.346	0.063	0.114	0.000	0.001	0.391
Galactosamine	0.087	0.016	0.061	0.015	0.106	0.013	0.129	0.023	0.132	0.190	0.015	0.205
**Galacturonic acid**	0.126	0.007	0.085	0.017	0.033	0.005	0.032	0.006	0.025	0.000	0.008	0.450
Hydroxylamine	0.364	0.082	0.257	0.057	0.245	0.050	0.266	0.067	0.154	0.122	0.460	0.402
**Hydroxybutyric acid**	0.061	0.011	0.036	0.009	0.023	0.009	0.053	0.013	0.052	0.010	0.160	0.043
Hydroxyproline	0.149	0.020	0.109	0.017	0.112	0.006	0.320	0.242	0.078	0.062	0.206	0.209
Glycolic acid	0.080	0.005	0.062	0.010	0.093	0.005	0.119	0.019	0.085	0.050	0.011	0.110
Glyceric acid	2.871	0.616	2.764	0.676	5.229	0.453	5.610	0.733	0.454	0.005	0.006	0.333
Glycerol	1.295	0.449	0.665	0.248	0.404	0.089	0.493	0.114	0.124	0.048	0.271	0.274
Glycerol-3-phosphate	0.084	0.016	0.070	0.012	0.067	0.006	0.068	0.012	0.236	0.165	0.458	0.462
Glycine	7.869	0.447	6.916	1.069	4.999	0.260	4.939	0.856	0.216	0.000	0.086	0.474
Glutamine	9.446	0.826	8.215	1.311	8.504	0.752	10.208	1.739	0.221	0.208	0.188	0.195
Glutamine, dehydro-	2.065	0.428	1.175	0.245	2.364	0.250	2.570	0.325	0.051	0.280	0.002	0.312

Metabolite	C	C, SE	CG	CG, SE	S	S, SE	SG	SG, SE	*P* value, C/CG	*P* value, C/S	*P* value, CG/SG	*P* value, S/SG
Glutamic acid	3.722	0.347	3.515	0.570	5.127	0.468	5.940	0.951	0.381	0.017	0.025	0.230
Glucose	24.564	12.310	17.594	8.497	85.897	20.676	98.047	29.795	0.325	0.015	0.016	0.372
Glucose-6-phosphate	1.004	0.059	0.841	0.152	1.398	0.033	1.513	0.206	0.172	0.000	0.011	0.299
Dihydrouracil	0.631	0.132	0.500	0.090	0.538	0.032	0.649	0.171	0.215	0.259	0.229	0.272
Iditol	9.986	4.636	5.631	2.826	4.569	2.174	0.902	0.596	0.220	0.160	0.071	0.074
Isoleucine	1.050	0.091	0.881	0.135	0.091	0.008	0.089	0.016	0.160	0.000	0.000	0.464
**Leucine**	1.322	0.126	0.846	0.207	0.096	0.026	0.106	0.024	0.037	0.000	0.004	0.398
Citric acid	1.142	0.250	0.853	0.203	1.485	0.158	1.685	0.213	0.193	0.137	0.007	0.232
Malonic acid	0.117	0.010	0.094	0.017	0.071	0.006	0.063	0.004	0.129	0.001	0.055	0.164
Maltose	0.160	0.008	0.123	0.021	0.063	0.005	0.068	0.010	0.064	0.000	0.020	0.338
Mannose	5.282	2.917	3.655	1.777	34.520	2.011	43.534	7.509	0.322	0.000	0.000	0.140
Melibiose	0.050	0.005	0.035	0.007	0.066	0.009	0.074	0.013	0.055	0.063	0.013	0.318
Myo-inositol	14.222	0.911	12.079	1.840	22.073	0.703	26.235	5.366	0.160	0.000	0.018	0.233
Lactic acid	0.301	0.101	0.246	0.073	0.211	0.022	0.381	0.153	0.333	0.206	0.223	0.154
Urea	0.081	0.009	0.064	0.013	0.022	0.003	0.025	0.005	0.159	0.000	0.012	0.307
2-Oxoglutaric acid	0.021	0.004	0.016	0.003	0.018	0.003	0.022	0.003	0.189	0.290	0.091	0.168
Oxoproline	9.276	0.589	7.709	1.326	11.211	0.323	12.038	1.237	0.153	0.009	0.016	0.268
**Ornithine**	0.193	0.018	0.162	0.032	0.029	0.003	0.022	0.002	0.200	0.000	0.002	0.028
Palmitic acid	2.327	0.437	1.722	0.277	1.991	0.198	2.084	0.547	0.134	0.251	0.284	0.438
Pyrophosphoric acid	23.635	2.382	18.239	3.867	16.855	2.069	18.517	2.653	0.129	0.027	0.477	0.315
Proline	0.861	0.043	0.749	0.113	0.387	0.032	0.418	0.095	0.191	0.000	0.021	0.383
Putrescine	2.732	0.235	2.121	0.382	2.190	0.274	2.597	0.508	0.100	0.080	0.234	0.248
Ribose	3.247	0.997	2.479	0.778	0.927	0.025	1.016	0.143	0.278	0.029	0.052	0.278
Sucrose	27.624	3.874	20.873	4.789	146.975	11.382	145.912	29.882	0.147	0.000	0.002	0.487
Serine	3.414	0.241	2.781	0.415	2.361	0.065	2.974	0.452	0.107	0.002	0.378	0.110
**Sinapic acid**	1.097	0.106	0.724	0.135	0.556	0.037	0.573	0.093	0.024	0.001	0.187	0.431
Trehalose	10.100	2.408	7.737	2.515	11.010	1.274	10.093	0.856	0.255	0.373	0.200	0.281
Threonine	2.384	0.184	1.899	0.272	1.252	0.078	1.416	0.209	0.083	0.000	0.091	0.240
Tryptophan	1.517	0.160	1.229	0.234	0.245	0.016	0.240	0.028	0.165	0.000	0.002	0.437
Phenylalanine	1.900	0.193	1.445	0.251	0.169	0.012	0.162	0.029	0.088	0.000	0.001	0.413
Phosphoric acid	1.121	0.220	0.914	0.217	0.122	0.015	0.143	0.037	0.258	0.002	0.005	0.299
Fructose	0.353	0.060	0.259	0.037	0.364	0.067	0.389	0.097	0.105	0.452	0.122	0.420
Ethanolamine	0.596	0.204	0.336	0.067	0.696	0.154	0.808	0.130	0.132	0.351	0.004	0.294
Malic acid	8.562	0.279	7.173	1.245	4.778	0.204	5.169	0.659	0.155	0.000	0.092	0.293
Succinic acid	0.363	0.018	0.333	0.067	0.281	0.037	0.366	0.053	0.337	0.040	0.354	0.110
