# Supplementary material for: Sucrose and Mannans Affect Arabidopsis Shoot Gravitropism at the Cell Wall Level
Source: Plants (Basel). 2024 Jan 11;13(2):209. doi: 10.3390/plants13020209 (PMC10819476; doi:10.3390/plants13020209)
Supplement: Supplementary file 1 [file plants-13-00209-s001.zip › Table S2.pdf]

**Table S2.** Relative content of metabolites in hypocotyls of gravistimulated and nongravistimulated wild type Col-0 and *csla2csla3csla9* Arabidopsis seedlings. Results of three independent experiments (n=8). Data are given in arbitrary units.
Abbreviations:
WT - nongravistimulated wild type Col-0 seedlings;
M - nongravistimulated *csla2csla3csla9* triple mutant seedlings;
WTG - gravistimulated wild type Col-0 seedlings;
MG - gravistimulated *csla2csla3csla9* triple mutant seedlings.
Data are means, standard errors (SE) and *P* values of pairwise comparisons of the above variants using Student's *t*-test.
All metabolites for which significant differences between nongravistimulated and gravistimulated variants (WT vs. WTG and M vs. MG) were found are indicated in bold. In the case of a significant increase in the content of a metabolite in gravistimulated vs. nongravistimulated seedlings (WTG vs. WT and MG vs. M), as well as in *csla2csla3csla9* triple mutant seedlings compared with wild type Col-0 seedlings (M vs. WT and MG vs. WTG) the respective *P* values are shown on a red background. In the case of a significant decrease in the content of a metabolite in the abovementioned comparisons the respective *P* values are shown on a blue background.

| **Metabolite** | **WT** | **WT, SE** | **WTG** | **WTG,SE** | **M** | **M, SE** | **MG** | **MG, SE** | ***P* value, WT/WTG** | ***P* value, WT/M** | ***P* value, WTG/MG** | ***P* value, M/MG** |
|---|---|---|---|---|---|---|---|---|---|---|---|---|
| 1-Pyrroline-3-hydroxy-5-carbonic acid | 3.300 | 0.681 | 3.573 | 0.301 | 3.241 | 0.299 | 3.457 | 0.558 | 0.361 | 0.469 | 0.429 | 0.371 |
| Alanine | 0.263 | 0.070 | 0.396 | 0.040 | 0.244 | 0.024 | 0.228 | 0.057 | 0.063 | 0.403 | **0.017** | 0.398 |
| β-Alanine | 0.138 | 0.028 | 0.145 | 0.012 | 0.141 | 0.014 | 0.153 | 0.024 | 0.415 | 0.466 | 0.385 | 0.336 |
| Alanyl-alanine | 0.599 | 0.089 | 0.575 | 0.142 | 0.519 | 0.064 | 0.598 | 0.133 | 0.445 | 0.238 | 0.454 | 0.303 |
| Asparagine | 0.209 | 0.050 | 0.257 | 0.029 | 0.194 | 0.031 | 0.193 | 0.047 | 0.211 | 0.402 | 0.139 | 0.496 |
| Aspartic acid | 0.128 | 0.028 | 0.131 | 0.009 | 0.169 | 0.016 | 0.199 | 0.031 | 0.456 | 0.116 | **0.037** | 0.208 |
| Benzoic acid | 0.038 | 0.005 | 0.052 | 0.008 | 0.041 | 0.004 | 0.035 | 0.004 | 0.077 | 0.340 | **0.044** | 0.202 |
| β-D-allose | 0.000 | 0.000 | 0.000 | 0.000 | 0.000 | 0.000 | 0.000 | 0.000 | 0.190 | 0.074 | 0.295 | 0.398 |
| 4-Aminobutanoic acid | 0.170 | 0.037 | 0.176 | 0.013 | 0.146 | 0.018 | 0.163 | 0.020 | 0.443 | 0.285 | 0.295 | 0.275 |
| Cellobiose | 0.000 | 0.000 | 0.027 | 0.027 | 0.000 | 0.000 | 0.000 | 0.000 | 0.174 | 0.413 | 0.174 | 0.408 |
| D230150 | 0.039 | 0.013 | 0.027 | 0.010 | 0.025 | 0.007 | 0.025 | 0.005 | 0.234 | 0.188 | 0.445 | 0.494 |
| Ethanolamine | 0.417 | 0.143 | 0.250 | 0.053 | 0.268 | 0.043 | 0.316 | 0.023 | 0.151 | 0.174 | 0.140 | 0.174 |
| Fumaric acid | 0.037 | 0.008 | 0.036 | 0.004 | 0.039 | 0.006 | 0.039 | 0.006 | 0.487 | 0.419 | 0.350 | 0.484 |
| Galactaric acid | 0.000 | 0.000 | 0.000 | 0.000 | 0.000 | 0.000 | 0.000 | 0.000 | 0.425 | 0.119 | 0.260 | 0.294 |
| Glycine | 1.170 | 0.234 | 1.198 | 0.110 | 1.194 | 0.100 | 1.148 | 0.194 | 0.457 | 0.463 | 0.414 | 0.419 |
| 2-Phenylglicine | 0.000 | 0.000 | 0.000 | 0.000 | 0.000 | 0.000 | 0.000 | 0.000 | 0.068 | 0.391 | 0.415 | 0.129 |
| Hexadecanoic acid | 0.791 | 0.117 | 0.870 | 0.076 | 0.879 | 0.088 | 0.854 | 0.093 | 0.290 | 0.279 | 0.446 | 0.424 |
| Hydroxylamine | 0.073 | 0.014 | 0.081 | 0.007 | 0.094 | 0.011 | 0.078 | 0.007 | 0.312 | 0.124 | 0.399 | 0.119 |
| Idose | 0.105 | 0.043 | 0.094 | 0.013 | 0.112 | 0.020 | 0.136 | 0.014 | 0.406 | 0.447 | **0.025** | 0.171 |
| Myo-inositol | 3.673 | 0.702 | 3.646 | 0.290 | 3.666 | 0.368 | 3.875 | 0.500 | 0.486 | 0.497 | 0.350 | 0.371 |
| Isoleucine | 0.232 | 0.056 | 0.240 | 0.027 | 0.215 | 0.026 | 0.229 | 0.048 | 0.449 | 0.398 | 0.424 | 0.404 |
| Leucine | 0.131 | 0.032 | 0.120 | 0.015 | 0.130 | 0.014 | 0.147 | 0.029 | 0.377 | 0.486 | 0.208 | 0.300 |
| Lysine | 0.000 | 0.000 | 0.000 | 0.000 | 0.000 | 0.000 | 0.000 | 0.000 | 0.409 | 0.319 | 0.114 | 0.229 |

| Metabolite | WT | WT, SE | WTG | WTG,SE | M | M, SE | MG | MG, SE | *P* value, WT/WTG | *P* value, WT/M | *P* value, WTG/MG | *P* value, M/MG |
|---|---|---|---|---|---|---|---|---|---|---|---|---|
| Lyxose | 0.120 | 0.049 | 0.091 | 0.034 | 0.072 | 0.017 | 0.082 | 0.013 | 0.320 | 0.191 | 0.406 | 0.321 |
| Malic acid | 0.020 | 0.004 | 0.020 | 0.001 | 0.018 | 0.003 | 0.023 | 0.003 | 0.496 | 0.341 | 0.202 | 0.131 |
| NA_aldehyde(C9H18O) | 0.628 | 0.121 | 0.691 | 0.090 | 0.762 | 0.109 | 0.920 | 0.171 | 0.341 | 0.212 | 0.133 | 0.227 |
| NA_benzyl ester | 1.244 | 0.300 | 1.447 | 0.268 | 1.064 | 0.140 | 1.111 | 0.372 | 0.311 | 0.299 | 0.239 | 0.455 |
| NA114002 | 0.763 | 0.153 | 0.907 | 0.119 | 0.777 | 0.090 | 0.639 | 0.185 | 0.236 | 0.470 | 0.125 | 0.260 |
| Octadecanoic acid | 1.099 | 0.180 | 1.096 | 0.072 | 1.182 | 0.137 | 1.054 | 0.117 | 0.493 | 0.360 | 0.385 | 0.247 |
| Ornithine | 0.000 | 0.000 | 0.001 | 0.000 | 0.001 | 0.000 | 0.001 | 0.000 | 0.271 | 0.132 | 0.257 | 0.413 |
| Phenylalanine | 0.172 | 0.043 | 0.188 | 0.025 | 0.160 | 0.021 | 0.165 | 0.033 | 0.380 | 0.405 | 0.295 | 0.456 |
| Phosphoric acid | 2.666 | 0.361 | 3.128 | 0.270 | 3.080 | 0.322 | 3.575 | 0.489 | 0.162 | 0.203 | 0.222 | 0.209 |
| Proline | 0.079 | 0.014 | 0.087 | 0.022 | 0.112 | 0.018 | 0.154 | 0.032 | 0.374 | 0.084 | 0.059 | 0.144 |
| **Carboxy-proline** | 0.002 | 0.001 | 0.001 | 0.001 | 0.000 | 0.000 | 0.000 | 0.000 | 0.209 | 0.093 | 0.180 | **0.050** |
| 4-Hydroxyproline | 0.161 | 0.024 | 0.166 | 0.010 | 0.127 | 0.012 | 0.124 | 0.012 | 0.420 | 0.118 | **0.008** | 0.425 |
| Psicose | 1.771 | 0.711 | 1.114 | 0.105 | 2.442 | 0.559 | 2.115 | 0.356 | 0.195 | 0.235 | **0.015** | 0.315 |
| Ribose | 0.310 | 0.088 | 0.260 | 0.018 | 0.386 | 0.057 | 0.387 | 0.057 | 0.296 | 0.242 | **0.035** | 0.491 |
| Serine | 0.142 | 0.027 | 0.143 | 0.010 | 0.163 | 0.015 | 0.168 | 0.025 | 0.486 | 0.251 | 0.189 | 0.434 |
| Similar to 2,3-dihydropropyl ester of octadecanoic acid | 3.152 | 0.502 | 3.211 | 0.217 | 3.937 | 0.646 | 3.278 | 0.415 | 0.459 | 0.177 | 0.445 | 0.204 |
| Sinapic acid | 0.041 | 0.008 | 0.043 | 0.005 | 0.044 | 0.007 | 0.056 | 0.009 | 0.411 | 0.370 | 0.116 | 0.174 |
| Sucrose | 3.115 | 0.689 | 3.387 | 0.395 | 3.318 | 0.468 | 4.063 | 0.683 | 0.369 | 0.406 | 0.206 | 0.194 |
| Allo-threonine | 0.291 | 0.062 | 0.308 | 0.031 | 0.305 | 0.033 | 0.328 | 0.061 | 0.405 | 0.427 | 0.391 | 0.373 |
| Tyrosine | 0.131 | 0.043 | 0.240 | 0.109 | 0.102 | 0.011 | 0.098 | 0.014 | 0.188 | 0.263 | 0.118 | 0.415 |
| Valine | 0.276 | 0.064 | 0.222 | 0.047 | 0.260 | 0.028 | 0.225 | 0.041 | 0.255 | 0.412 | 0.481 | 0.251 |
